# An Efficient Multi-Objective White Shark Algorithm

**DOI:** 10.3390/biomimetics10020112

**Published:** 2025-02-13

**Authors:** Wenyan Guo, Yufan Qiang, Fang Dai, Junfeng Wang, Shenglong Li

**Affiliations:** School of Science, Xi’an University of Technology, Xi’an 710048, China; 2210920036@stu.xaut.edu.cn (Y.Q.); daifang@xaut.edu.cn (F.D.); 18829537964@163.com (J.W.); 2230920018@stu.xaut.edu.cn (S.L.)

**Keywords:** White Shark Optimization algorithm, multi-objective optimization, non-dominated sorting, elite reservation, subway tunnel foundation pits optimization

## Abstract

To balance the diversity and stringency of Pareto solutions in multi-objective optimization, this paper introduces a multi-objective White Shark Optimization algorithm (MONSWSO) tailored for multi-objective optimization. MONSWSO integrates non-dominated sorting and crowding distance into the White Shark Optimization framework to select the optimal solution within the population. The uniformity of the initial population is enhanced through a chaotic reverse initialization learning strategy. The adaptive updating of individual positions is facilitated by an elite-guided forgetting mechanism, which incorporates escape energy and eddy aggregation behavior inspired by marine organisms to improve exploration in key areas. To evaluate the effectiveness of MONSWSO, it is benchmarked against five state-of-the-art multi-objective algorithms using four metrics: inverse generation distance, spatial homogeneity, spatial distribution, and hypervolume on 27 typical problems, including 23 multi-objective functions and 4 multi-objective project examples. Furthermore, the practical application of MONSWSO is demonstrated through an example of optimizing the design of subway tunnel foundation pits. The comprehensive results reveal that MONSWSO outperforms the comparison algorithms, achieving impressive and satisfactory outcomes.

## 1. Introduction

**Figure 1:**
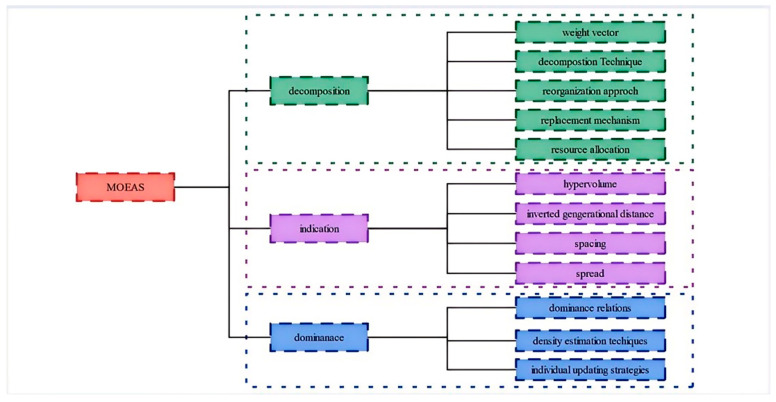
Detailed description of MOEAs.

In the domain of multi-objective (MO) optimization, researchers are tirelessly pursuing more efficient methodologies to tackle these intricate challenges, aiming to procure sets of Pareto optimal solutions (POS) that meticulously mirror the genuine Pareto front (PF). The recent advancements in MO Evolutionary Algorithms (MOEAs) can be primarily categorized into three distinct groups: (1) decomposition-based MOEAs, (2) indicator-based MOEAs, and (3) those grounded in Pareto dominance-based principles. The detailed description of MOEAs is shown in Figure 1.

(1) Decomposition-based MOEAs

The core idea behind MOEAs based on decomposition, known as MOEA/D, involves employing a set of well-distributed weight vectors within the objective space to convert the intricate multi-objective optimization problem into a sequence of simpler, more manageable sub-problems. By leveraging the interrelations among these sub-problems, the algorithm computes objective function values, which function as fitness indicators, ultimately culminating in a comprehensive solution set. Research endeavors concerning this algorithm can be encapsulated into five pivotal domains [1]: (1) weight vector design, (2) decomposition technique, (3) reorganization approach, (4) replacement mechanism, and (5) computational resource allocation framework.

(1) Weight vectors (WCs) play a pivotal role in ensuring population diversity within decomposition-based MOEAs. Research endeavors in this realm primarily concentrate on two vital facets: the generation of uniformly distributed WCs and methodologies for adaptive WCs generation. The decomposition-based MO optimization method MOEA/D, initially proposed by Zhang et al. [2], utilizes the simplex method to generate uniformly distributed WCs. Building upon this foundation, Tan et al. [3] further refined the MOEA/D by introducing the UDEM algorithm, which surpassed the simplex lattice method in achieving a more balanced distribution of vectors within the objective space. Ma et al. [4] then introduced an even more adaptive approach in the objective space, seamlessly blending the UDEM and simplex mesh design methods to create alternative WCs. Harada et al. [5] contributed by offering a method to adaptively augment the WCs for particularly challenging sub-problems. Li et al. [6] took a novel route by introducing a technique that automatically recognizes the state of vectors corresponding to individual sub-problems, thereby generating weight vectors for each sub-problem while anticipating the computational complexity associated with each. More recently, Qi et al. [7] suggested an innovative two-stage strategy to fine-tune the generation of WCs.

(2) Decomposition-based methods encompass the Weighted Sum, Tchebycheff, and Penalty-based Boundary Intersection approaches. Given the unique attributes of diverse optimization problems, traditional decomposition methods necessitate specific enhancements. Sato [8] put forth the innovative inverted Penalty-based Boundary Intersection method. Jiang et al. [9] introduced two functions, MSF and PBSF, to dynamically adjust the extent of the decomposition region. Liu et al. [10], meanwhile, integrated the MOEA/D framework to tackle decomposition problems. This method not only dynamically adjusts the region’s size but also significantly boosts the diversity of the algorithms.

(3) The reorganization strategy primarily aims to enhance parent selection and devise novel methods for generating offspring, tailored to the characteristics of the evolutionary process. Wang et al. [11] seamlessly integrated evolutionary operators to bolster the effectiveness and diversity of the original method. Ke et al. [12] harmoniously united MOEA/D with ACO, leveraging the strengths of both global and local information to optimize a suite of MO sub-problems.

(4) The replacement strategy is designed to mitigate the risk of losing population diversity when a new solution supplants an existing one. Typically, algorithms strike a balance between diversity and convergence by imposing constraints on the replacement scope of new solutions and meticulously selecting promising candidates to pair with their corresponding sub-problems. As an illustration, Li et al. [13] seamlessly integrated the NDS into the MOEA/D framework, adopting a Pareto dominance-based replacement strategy.

(5) This strategy allocates computational resources to the more challenging sub-problems by evaluating the difficulty of each sub-problem’s solution, thereby enhancing the discovery of superior solutions while optimizing resource utilization. Cai et al. [14] utilize both the NDS and decomposition-based methods to maintain a steady count of internal and external populations, determining the probability of resource allocation to each sub-problem based on the number of individuals within it.

(2) Indicator-based MOEAs

The fundamental principle of indicator-based MOEAs is to sieve through individuals, selecting those suitable for continued iteration based on specific performance indicators, thereby guiding the population towards evolving higher-quality solutions. For instance, the HypE algorithm [15] employs the hypervolume (HV) metric to filter individuals, whereas the MaOEA/IGD algorithm [16] utilizes the Inverted Generational Distance (IGD) metric for similar purposes. Although the HV metric is widely used in low-dimensional problems, its computational complexity increases dramatically with the number of objectives and iterations. Consequently, there is a pressing need for algorithms capable of swiftly computing HV metrics. However, significant advancements in reducing HV complexity remain elusive.

(3) Domination-based MOEAs

In MO problems characterized by conflicting objectives, the term “dominance” is utilized to assess solution quality. The core concept of dominance-based MOEAs involves utilizing dominance relationships to stratify the population into distinct levels, where individuals within the same level exhibit no dominance over each other, thereby necessitating the selection of individuals through various mechanisms. Research on tackling MO problems through non-dominated sorting is pivotal for subsequent investigations and can be broadly classified into three primary areas: (1) dominance relations, (2) density estimation techniques, and (3) individual updating strategies.

(1) Traditional dominance relations exhibit varied performance across diverse problems, prompting the proposal of numerous refined dominance relations aimed at enhancing the efficiency of MO problem solutions. Yang et al. [17] introduced grid dominance, which boosts the dominance probability of individuals by segmenting the objective space into hypergrids. Adaptive fuzzy domination [18] redefined a robust dominance relation by incorporating fuzzy logic principles. Yuan and Elarbi, alongside others [19], integrated the concept of weight vectors from decomposition algorithms into dominance-based MOEAs, presenting hybrid dominance and RP [20] domination. Tian et al. [21] unveiled a novel non-dominated sorting method based on the target vector perspective, termed SDR dominance. Zitzler et al. [22] introduced the SPEAII algorithm, while Chalabi et al. [23] revealed a GMOMPA framework grounded in epsilon dominance.

(2) These strategies are utilized to select individuals within the same classification, ensuring that solutions with the greatest potential for development are preserved in each iteration. However, traditional density estimation strategies, such as the CD method, run the risk of allowing individuals situated further from the PF to persist, potentially compromising convergence. To overcome these limitations, Adra et al. [24] employed a Diversity Management operator to bolster algorithm diversity, striking a balance between convergence and diversity. Li et al. [25] introduced a novel Solution Distribution Estimation approach to fine-tune solution positions in the objective space, further enhancing solution performance.

(3) This area has witnessed numerous proposals and enhancements in MO optimization algorithms. Many researchers have substituted the genetic algorithms in NSGA-II with newer swarm intelligence algorithms that exhibit superior solving capabilities, embedding these algorithms into MO frameworks to augment both convergence and diversity. For instance, Hancer et al. [26] innovated a Pareto-based MO Artificial Bee Colony (MOABC) algorithm, incorporating an archive mechanism for characteristic selection. Abdallahi et al. [27] introduced a MO Efficient Symbiotic Organism Search (MOESOS) algorithm, applied to task scheduling optimization. Houssein et al. [28] developed a MOSMA that integrates the SMA with Pareto dominance and crowding distance. Khishe et al. [29] presented an MOChOA, demonstrating a superior capacity to evade local optima across diverse benchmark problems.

MO optimization algorithms stand at the vanguard of computational intelligence, diligently seeking solutions that concurrently optimize multiple, often conflicting objectives. Despite their proven effectiveness, there remain several avenues for further refinement. One critical area is enhancing their convergence properties to guarantee that the discovered solutions are closer to the genuine Pareto front. Another essential aspect is improving the diversity of solutions, thereby ensuring a more extensive and comprehensive coverage of the solution space.

In 2022, Malik Braik et al. [30] introduced the White Shark Optimizer (WSO), a novel meta-heuristic approach inspired by the feeding behavior of white sharks. This method encompasses four distinct phases: advancing towards prey, surrounding optimal targets, converging on sharks, and mimicking fish schooling behavior. While the WSO exhibits robust global search capabilities, it also encounters challenges, including limited computational accuracy and a propensity for premature convergence.

To facilitate MO intelligent optimization, an approach for solving MO optimization problems is presented by integrating MO thinking with the WSO. The key highlights of this article are as follows:

- Introduces a MONSWSO solution framework based on NSGA-II. The WSO boasts impressive exploration and development capabilities. By integrating the WSO with an elite non-dominated sorting (NDS) mechanism and a Pareto archive, the MONSWSO was developed. This novel method exhibits enhanced robustness and more efficient search capabilities.
- By incorporating a chaotic reverse initialization learning strategy, we generate a more diverse initialization population. Additionally, an adaptive evolution design is introduced to enhance local exploitation capabilities. Furthermore, a hybrid escape energy vortex fish aggregation strategy is utilized to promote the exploration of potential regions.
- Through a series of case studies with varying characteristics, including 23 MO benchmark functions and 4 MO engineering optimization problems, the performance of MONSWSO is rigorously verified through the analysis of five key measures. A practical MO optimization example, such as the optimal setup of an underpass tunnel above a pit, is presented to demonstrate the reliability of MONSWSO’s ability to tackle real-world problems.

The analytical conclusions affirm that the algorithm excels in handling a wide range of benchmarking and Multi-Objective (MO) engineering problems, achieving a Pareto Front (PF) with superior agglomeration and diversity. Comparative results highlight that MONSWSO demonstrates advantages in the majority of case studies and tends to surpass other comparative methods in performance.

Section 2 provides the definitions pertinent to this study. Section 3 elaborates on the WSO and the proposed MONSWSO in detail. Section 4 presents the results of MONSWSO in benchmarking problems. Following this section, the calculation results and analysis of MO engineering problems utilizing MONSWSO are described. Finally, the paper concludes with a summary. We hereby expertly declare that the abbreviations utilized within this article, along with their corresponding meanings, have been meticulously listed in Table A1 in Appendix A.

## 2. Related Concepts

For the convenience of narration, this section presents the relevant concepts and technologies needed in this paper.

### 2.1. MO Optimization

The standard MO optimization includes at least two conflicting targets that must be optimized simultaneously. A typical MO minimization can be expressed as Equation (1):

(1) $$\begin{array}{l} \min\;F(x)=\left(F_{1}(x),F_{2}(x),\ldots ,F_{m}(x)\right)\\ s.t.\;\left\{\begin{array}{l} GB_{k}(x)\leq 0,k=1,2,\ldots ,K\\ HE_{j}(x)=0.j=1,2,\ldots ,J\\ lo_{i}\leq x_{i}\leq up_{i},i=1,2,\ldots ,D \end{array}\right. \end{array}$$

where x denotes a decision variable with D dimension, F(x) denotes an objective vector with m dimension, $GB_{k}(x)$ denotes the k-th inequality constraint, $\;HE_{j}(x)$ denotes the j-th equality constraint.

For the MO problem, the prevailing research approach currently involves acquiring the POS set through Pareto domination and illustrating its distribution via the PF, which is defined as follows:

**Definition** **1.** *Pareto domination. If *$x_{A}$* and $x_{B}$ are two solutions of MO problem satisfying the constraints, if $F_{i}\left(x_{A}\right)\leq F_{i}\left(x_{B}\right)$ for all targets and there is at least one goal that satisfies $f_{j}\left(x_{A}\right)<f_{j}\left(x_{B}\right)$. At this point, it is called $x_{A}$ Pareto domination $x_{B}$ and is noted as such $x_{A}≺x_{B}$*.

**Figure 2:**
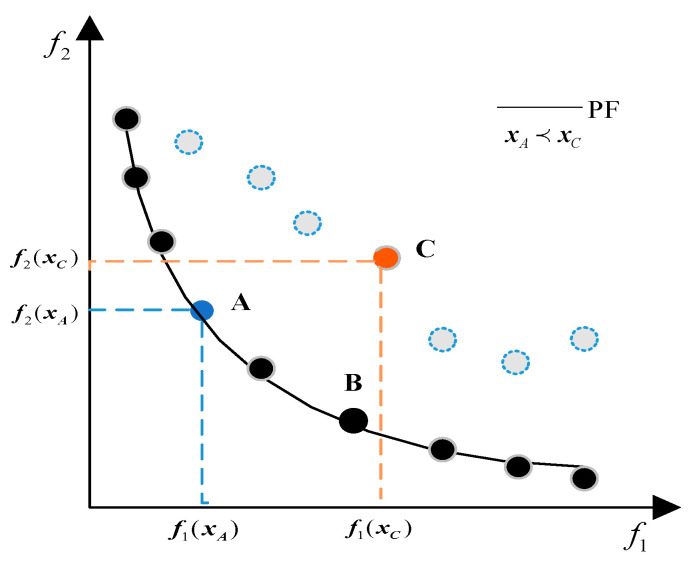
Graphical representation of related terms.

Figure 2 provides an intuitive depiction of the pertinent concepts in the bi-objective scenario. In the target space comprising imaginary and real points, for any imaginary point C associated with a particular solution $x_{C}$, at least one real point A corresponding to a feasible solution $x_{A}$ can be identified such that $x_{A}≺x_{C}$. Furthermore, for any two real points A and B linked to their respective solutions $x_{A}$, $x_{B}$, neither dominates the other. Consequently, the curves traced by connecting these real points constitute the PF.

**Definition** **2.** *POS. For any solution, $x^{\prime }$ in the feasible domain of the MO problem, $x≺x^{\prime }$ comes into existence and is known as the POS. The set of all POS is called the set of POS, and the collection of objective vectors formed by the elements in the set of POS is called the PF, i.e., PF = $\left\{F\left(x\right)\left|x\in \begin{array}{cc} \mathrm{The} & \begin{array}{cc} \mathrm{set} & \mathrm{of} \end{array} \end{array}POS\right.\right\}$*.

### 2.2. NDS and CD

**Figure 3:**
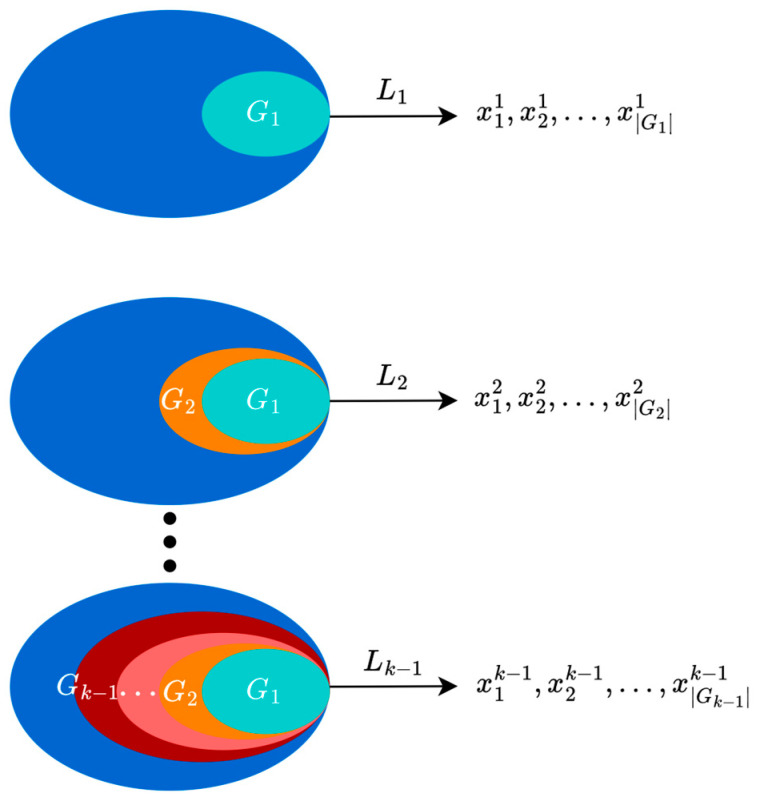
Schematic diagram of NDS.

This section elucidates the rapid NDS method [31] utilized for individual hierarchical classification, along with the CD calculation employed for individual merit selection in this text. Given a group G of individuals numbered N, the POS set within this group is identified $G_{1}$. Initially, individuals in the $G_{1}$ are placed in the first layer $L_{1}$. Subsequently, the individuals within the POS set in $G-G_{1}$ are ranked in the second layer $L_{2}$, and this process continues iteratively, with the remaining ND solutions being ranked in subsequent layers up to the t-th layer $L_{t}$, thereby completing the ranking of all individuals in G. An intuitive graphical representation of the NDS process is depicted in Figure 3.

For NDS populations, solutions occupying higher ranks are deemed superior to those in lower ranks. When it comes to comparing the superiority of solutions among individuals within the same rank, numerous literature sources have adopted the CD as a criterion. This metric assesses the relative superiority of two individuals within the same rank, with a larger CD indicating a better solution. The CD of the τ-th solution $x_{\tau }^{i}$ in $L_{i}$ is evaluated as shown in Equation (2)

(2) $$d_{L_{i}}^{\tau }=\sum_{j=1}^{m}\frac{\left|f_{j}^{\tau -1}-f_{j}^{\tau +1}\right|}{f_{j\max}-f_{j\min}},\tau =1,2,\ldots .\left|G_{i}\right|$$

where $F_{j\max}$ and $F_{j\min}$ are the maximum values of the j-th target in G. $F_{j}^{\tau -1}$ and $F_{j}^{\tau +1}$ denote the two targets of the j-th target neighboring $F\left(x_{\tau }^{i}\right)$ in the target space.

**Figure 4:**
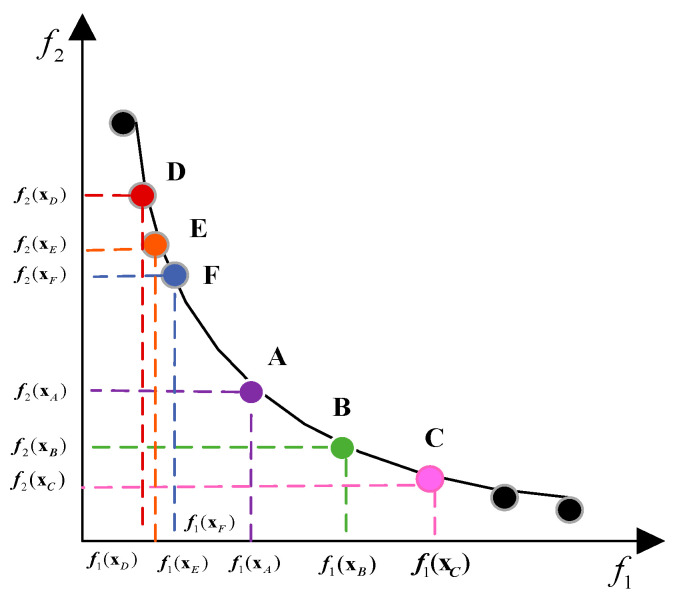
Schematic diagram of crowding distance.

A larger value of $d_{L_{i}}^{\tau }$ indicates that there are fewer points of interest in the vicinity of the point in the target space corresponding to $x_{\tau }^{i}$, suggesting that positions farther away from $x_{\tau }^{i}$ in the target space are not easily replaceable. Consequently, such positions hold greater significance and are thus more optimal. Figure 4 provides a visualization of the congestion distance, which demonstrates that point B has a greater congestion distance than point E. Therefore, point B is considered preferable.

### 2.3. Elite Retention Strategies

**Figure 5:**
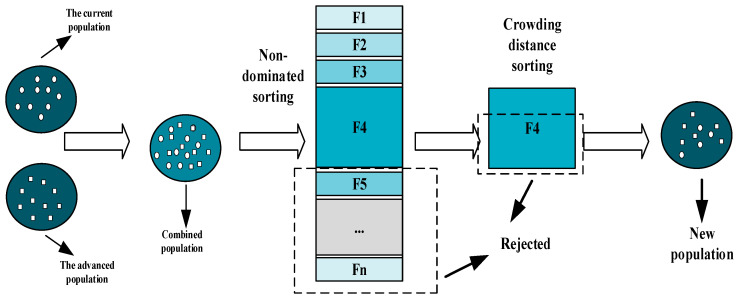
Elite selection process.

To accelerate the rate of population evolution and elevate the quality of the final PF, the newly formed group from each iteration is integrated with the existing group to form a combined population, which is subsequently filtered. Initially, this merged population undergoes rapid NDS to establish its hierarchical structure. Following this, the CD for each solution is calculated. Based on this CD information, a selection strategy is implemented to retain individuals, where N signifies the designated population size. These carefully selected individuals continue to evolve and adapt until the algorithm reaches its conclusion. Figure 5 provides a clear illustration of the elite retention strategy in action.

## 3. Multi-Objective White Shark Algorithm

To enhance the search capabilities of the WSO algorithm, this section introduces an enhanced version of WSO, built upon the foundation of the basic WSO algorithm and a thorough analysis of its shortcomings. By integrating this improved WSO into an MO framework, we propose a novel target-seizing optimization algorithm MONSWSO.

### 3.1. WSO

In 2022, Malik Braik and his colleagues introduced a novel meta-heuristic approach named WSO, which mimics the feeding behavior of white sharks on prey across four distinct phases: advancing toward the prey, surrounding the most promising target, converging toward fellow sharks, and exhibiting schooling behavior. This method boasts a potent global search capability.

#### 3.1.1. Move Towards the Quarry

When chasing prey, the white shark moves towards the prey in a fluctuating manner, as shown in Equation (3):

(3) $$v_{k+1}^{i}=\mu \left[v_{k}^{i}+p_{1}c_{1}\left(X_{gbest_{k}}-X_{k}^{i}\right)+p_{2}c_{2}\left(X_{best}^{v^{i}}-X_{k}^{i}\right)\right]$$

where i=1,…,N, $X_{gbest_{k}}$ is the best solution obtained, $X_{k}^{i}$ is the i-th solution in the k-th iteration, $X_{best}^{v^{i}}$ denotes the best solution passed by the i-th individual in the population, $c_{1}$ and $c_{2}$ are belongs to $\left[0,1\right]$, μ=0.352. $p_{1}$ and $p_{2}$ denotes the influence of $X_{gbest_{k}}$ and $X_{best}^{v^{i}}$, respectively. $p_{1}$ and $p_{2}$ is calculated as follows:

(4) $$p_{1}=p^{ma}+\left(p^{ma}-p^{mi}\right)\times e^{-{\left(4k/K_{\max}\right)}^{2}}$$

(5) $$p_{2}=p^{mi}+\left(p^{ma}-p^{mi}\right)\times e^{-{\left(4k/K_{\max}\right)}^{2}}$$

where k and $K_{\max}$ denote the current and final of iterations, $p^{mi}$ and $p^{ma}$ denote the minimum and maximum speeds, $p^{mi}=0.5$ and $p^{ma}=1.5$.

#### 3.1.2. Surrounding the Best Prey

White sharks gather information through their hearing and sense of smell, continuously adjusting their positions to seek out potential prey. The updates to their positions are governed by an adaptive switching probability, which escalates with the number of iterative follow-alongs or frequency updates, specifically.

(6) $$X_{k+1}^{i\left(1\right)}=\left\{\begin{array}{ll} X_{k}^{i}, & rand<mv_{k}\\ X_{k}^{i}+(1/h)\cdot v_{k+1}^{i}, & rand\geq mv_{k} \end{array}\right.$$

where h=0.8992 is the frequency of wave motion and the expression for $mv_{k}$ is

(7) $$mv_{k}=\frac{1}{\left(6.25+e^{\left(K_{\max}/2-k\right)/0.01}\right)}$$

#### 3.1.3. Moving Closer to the Best Sharks

During foraging, white sharks utilize both visual and olfactory cues to zero in on the most promising individuals, thereby enhancing their chances of capturing prey. They subsequently update their positions accordingly.

(8) $$X_{k+1}^{i\left(2\right)}=\left\{\begin{array}{ll} X_{gbest_{k}}+r_{1}D_{i}\mathrm{sgn}\left(r_{2}-0.5\right), & \;r_{3}<s_{s}\left(a\right)\\ X_{k+1}^{i\left(1\right)}, & otherwise\left(b\right) \end{array}\right.$$

where $D_{i}$ is the gap between the self and optimal value with the following expression and $\mathrm{sgn}\left(r_{2}-0.5\right)$ is a sign function.

(9) $$D_{i}=\left|r_{4}\times \left(X_{gbest_{k}}-X_{k+1}^{i\left(1\right)}\right)\right|$$

(10) $$s_{s}=\left|1-e^{\left(\frac{-0.0005\cdot k}{K_{\max}}\right)}\right|$$

#### 3.1.4. Cluster Behavior

For the position updated through Equations (6) and (8), the white shark determines its final location based on the clustering behavior.

(11) $$X_{k+1}^{i}=\frac{X_{k+1}^{i\left(2\right)-1}+X_{k+1}^{i\left(2\right)}}{2r_{5}}$$

where $r_{5}\in [0,1]$. The population is updated cyclically by Equations (1)–(11) to obtain the ideal solution to the problem.

### 3.2. MONSWSO

In the realm of MO optimization, researchers strive to balance both solution diversity and algorithm convergence. A uniform distribution of solutions is crucial in maintaining diversity within an MO context. Next, we introduce an enhanced version of WSO, named MONSWSO, seamlessly integrated into the framework of MO algorithms.

#### 3.2.1. Improved WSO

WSO employs a stochastic initial value strategy, resulting in a weak initialization population distribution that hinders the algorithm’s otherwise efficient search capabilities. When Equation (8) is utilized for development based on visual and olfactory intensity $s_{s}$, an excessively small $s_{s}$ value can lead to underdevelopment in WSO and a sluggish convergence rate. To address these two shortcomings of WSO, the MONSWSO algorithm is introduced, leveraging chaotic reverse initialization, adaptive evolution, and the vortex effect to enhance WSO’s performance. Subsequently, we propose a MONSWSO based on an improved WSO. The framework of this study mirrors that of NSGAII, with the main process comprising NDS, CD assignment, and elite sorting. In this algorithm, a more diverse initialized population is generated through a chaotic inverse initialization learning strategy, while an adaptive evolution strategy is introduced to facilitate local exploitation of potential areas. Furthermore, a hybrid escape energy mechanism and vortex fish aggregation strategy are employed to bolster the exploitation of potential regions and prevent the algorithm from falling into local optima.

(1) Chaotic reverse initialization

The inherent convenience and stochastic nature of chaos enable it to generate more homogeneous solutions [32], while reverse learning [33] techniques can produce potential solutions that are closer to the optimal one. By integrating these two concepts into the initialization process of WSO, a high-quality initial population can be obtained. Tent Chaos, in particular, exhibits excellent uniformity and swift iteration speed. Its evolution process is as follows:

(12) $$C_{s+1}=\left\{\begin{array}{ll} 2C_{s} & ,C_{s}<0.5\\ 2\left(1-C_{s}\right) & ,C_{s}\geq 0.5 \end{array}\right.$$

For an optimization problem involving a population size of *N* and a decision space of *D* dimensions, the Tent mapping is initially utilized to generate N×D points, which are then arranged into a chaotic matrix $C={\left(C_{i,d}\right)}_{N\times D}$. Subsequently, the *d*-th dimension of the *i*th individual within the population is determined by:

(13) $$X_{0}^{i,d}=l_{d}+C_{i,d}\left(u_{d}-l_{d}\right),\;\left(i=1,2,\ldots ,N,d=1,2,\ldots ,D\right)$$

(14) $$OX_{0}^{i,d}=lo_{d}+up_{d}-X_{0}^{i,d}$$

For the chaotic population $G_{0}={\left(X_{0}^{i,d}\right)}_{N\times D}$ and the inverse population $OG_{0}={\left(OX_{0}^{i,d}\right)}_{N\times D}$, N individuals were selected to form the starting group according to the selection strategy.

(2) Adaptive evolution and vortex effects

In WSO, the development phase relies heavily on probabilistic factors $s_{s}$. When the parameter $s_{s}$ is too small, the algorithm adopts fewer update modes during the execution of Equation (8a), leading to insufficient development ability and a slowdown in convergence speed. To address this, we incorporate a parameter E defined by an exponential decay function [34] with stochastic properties. This allows us to design diverse update modes for the development phase, thereby balancing the algorithm’s exploration and development capabilities. The defining equation for [35,36,37] is as follows:

(15) $$E_{k}=4E_{r}\cdot r_{6}\cdot e^{-\frac{1.5-k}{K_{\max}}}$$

where $E_{r}$ is a randomly given value on $\left[-1,1\right]$ and $r_{6}$ is a random number on $\left[0,1\right]$.

When $\left|E_{k}\right|>1$ the prey possesses greater escape energy and is situated farther from the target, incorporating the elite-guided forgetting mechanism can help the group explore innovative directions. This enhances the global leading capability during the development process, at which point Equation (8) is updated to:

(16) $$X_{k+1}^{i}=\left\{\begin{array}{ll} \left(\omega -\ln\left(r_{7}\right)\right)\cdot X_{gbest_{k}}+r_{1}\cdot D_{i}\cdot \mathrm{sgn}\left(r_{2}-0.5\right), & r_{3}<s_{s}\\ X_{k+1}^{i\left(1\right)}, & r_{3}\geq s_{s} \end{array}\right.$$

where the adaptive evolution factor is given in the following equation:

(17) $$\omega =e^{-0.8\cdot \ln\left(1+\frac{10\cdot k}{K_{\max}}\right)}$$

When $\left|E_{k}\right|\leq 1$, the prey is close to the target and the use of random wandering and vortex effects helps the agent jump out of the trough in order to obtain a better solution, specifically:

(18) $$X_{k+1}^{i\left(3\right)}=X_{gbest_{k}}+0.5\cdot {\left(1-\frac{k}{K_{\max}}\right)}^{\left(\frac{2-k}{K_{\max}}\right)}\cdot \left[U_{L}⊗\left(U_{L}⊗X_{gbest_{k}}-X_{k+1}^{i\left(1\right)}\right)\right]$$

where $U_{L}$ is the vector of Levy [38] distribution and ⊗ denotes the point-to-point multiplication of two vectors.

The vortex effect is updated as:

(19) $$X_{k+1}^{i}=\left\{\begin{array}{l} X_{k+1}^{i\left(3\right)}+{\left(1-\frac{k}{K_{\max}}\right)}^{\left(2-\frac{k}{K_{\max}}\right)}\cdot \left[l+r_{9}⊗\left(u-l\right)\right]⊗U^{i}.r_{8}\leq 0.2\\ X_{k+1}^{i\left(3\right)}+\left[0.2\cdot (1-r_{10})+r_{11}\right]\cdot \left(X_{k+1}^{r_{t1}}-X_{k+1}^{r_{t2}}\right).\;r_{8}>0.2 \end{array}\right.$$

where $X_{k+1}^{r_{t1}}$, $X_{k+1}^{r_{t2}}$ are two random solutions of group composed of a $X_{k+1}^{i\left(3\right)}$, and $U^{i}$ is a selection function, specified as:

(20) $$U^{i}=\left\{\begin{array}{l} 0,\;r_{10}\leq 0.2\\ 1,\;r_{10}>0.2 \end{array}\right.$$

where $r_{8}$, $r_{9}$, $r_{10}$, $r_{11}$ is a random number on $\left[0,1\right]$.

#### 3.2.2. Multi-Objective WSO Algorithm

The WSO algorithm, enhanced with chaotic reverse initialization, adaptive evolution, and vortex effect improvements, is designated as the MONSWSO. This algorithm significantly boosts the optimization performance by utilizing a high-quality initialized population and an evolutionary strategy that balances both exploration and exploitation. By integrating it with the MO framework, we propose the WSO-based MO algorithm, also known as MONSWSO.

The cornerstone of MO intelligent algorithms lies in the comparison of solution merits and the distribution of solution diversity. The MONSWSO algorithm enhances both the distribution and quality of solutions, ensuring a superior performance in addressing MO optimization problems.

- The most optimal individual $X_{gbest_{k}}$ of the population $G_{k}$ in generation k is selected by NDS of $G_{k}$ and randomly selecting one of the individuals in the first tier $L_{t}$ as $X_{gbest_{k}}$.
- The principle of initial population selection is to merge the chaotic initialized population and the inverse population, select N individuals from the elite non-dominated ordering of the resulting 2N individuals to form the initial population $G_{0}$, and record the optimal individuals. $X_{gbest_{0}}$

The iterative process of MONSWSO is listed in Algorithm 1.

**Algorithm 1:** The iterative process of MONSWSO Input: N, D, $K_{\max}$, $G_{0}$
Output: $G_{K}$
1:     Select $G_{0}$, $X_{gbest_{0}}$ according to Equations (11)–(13) 2:     **While**
$k<K_{\max}$ **do** 3:     Update ${\mathrm{pop}}_{1}=G_{k}$
4:     Update $mv_{k}$, $s_{s}$, $E_{k}$, $\omega_{k}$
5:     **For**
i=1 to N
6:       Use Equations (3)–(6) to renew solutions7:     **End for**8:     **For**
i=1 to N
9:       **If**
$\left|E_{k}\right|>1$ 10:   Update individuals according to Equation (16) 11:  **Else** 12:   Update individuals according to Equations (17) and (18) 13:  **End if** 14: **End for**15: Combine $G_{k}$ and ${\mathrm{pop}}_{1}$
16: Sort the combined group with the elitist NDS and find N excellent individuals17: k=k+1
18: **End while**19: Obtain the optimal population

MONSWSO employs a chaotic reverse initialization strategy to enhance the uniformity of population distribution and the adaptability of solutions. The approach of merging groups and selecting the final updated population through elite NDS and diversity retains high-quality solutions while bolstering solution diversity.

Furthermore, MONSWSO incorporates an energy switching strategy and selects an adaptive evolutionary path, enabling the group to swiftly converge towards the PF of the problem. This is facilitated by random wandering and vortex effects, which augment the algorithm’s exploration of potential solution regions.

## 4. Numerical Simulations

The superiority of MONSWSO is demonstrated through three representative types of MO problems: 23 well-known MO functions, 4 engineering problems, and the optimization challenge of subway tunnel pit excavation.

### 4.1. Experimental Setting

Table 1 details the 23 MO problems, while Table 2 outlines the characteristics of the 4 engineering design problems. In the experiments, five leading and advanced multi-objective algorithms—NSGAII, PESAII, MOPSO, MOALO, and MOGWO—are selected for comparison with classical test functions. For the engineering examples, NSGAII, PESAII, MOPSO, IBEA, and SMPSO are chosen, with parameter settings N=300, $K_{\max}=300$ consistent with their original literature. The algorithms are independently run 10 times, and the mean (M) and standard deviation (Sd) of comparison indices are calculated for analysis. IGD [39], Spacing, Spread and HV [40] four comparison metrics are used to measure the advantage of the algorithms.

### 4.2. Multi-Target Testing Experiments

This subsection presents the experimental results of MONSWSO on 11 two-objective test problems and 12 three-objective test problems and analyzes the superiority of MONSWSO from a statistical perspective.

#### 4.2.1. The Two-Objective Test Problem

Table 3 presents the M and Sd of all metrics and test results for MONSWSO, NSGAII, PESAII [41], MOPSO [42], MOALO [43], and MOGWO [44] on the two-objective benchmark test. The bold is to emphasize the best value. In terms of convergence accuracy, MONSWSO’s results surpass the comparison algorithms on the majority of test functions, with the exception of DEB1, FON2, and LAU, which exhibit slightly inferior performance compared to NSGAII. This indicates that MONSWSO possesses exceptional convergence capabilities.

The Spacing index results clearly indicate that our algorithm outperforms all other comparative algorithms across the entire spectrum of test functions, with the sole exception of ZDT6, where its performance is marginally inferior to NSGA-II. This underscores the exceptional uniformity of the solutions derived by MONSWSO. Furthermore, the Spread metric attests to MONSWSO’s outstanding performance across all test functions, highlighting its impressive breadth.

Regarding the HV metrics, while MONSWSO’s results for ZDT1 are inferior to NSGAII and those for ZDT3 lag behind PESAII, MOALO, and MOGWO, it nonetheless achieves excellent results on all other functions. This demonstrates that the algorithm possesses strong overall performance.

The Wilcoxon rank sum test was utilized to evaluate the differences between MONSWSO and other algorithms. The symbol ‘−’ signifies that MONSWSO surpasses the comparative algorithm. For the test problems that encompass the IGD, Spacing, Spread, and HV metrics. In terms of the IGD metric, all metric values significantly diverge, with the exception of NSGA-II and MOALO, which demonstrated superior performance compared to MONSWSO. Regarding the Spacing metric, all metric values significantly differ from the proposed algorithm, except for PESAII, whose results underperformed those of the comparative algorithms. Notably, PESAII’s results on ZDT6 and MOPSO’s results on ZDT4 showed no significant difference from MONSWSO’s. For the Spread metric, only the results for ZDT4 and ZDT6 did not significantly differ from those obtained by NSGA-II, while all other results exhibited significant differences. Concerning the HV metrics, no significant difference was observed between MONSWSO and PESAII. However, all other algorithms demonstrated significant differences when compared to the proposed algorithm.

**Figure 6:**
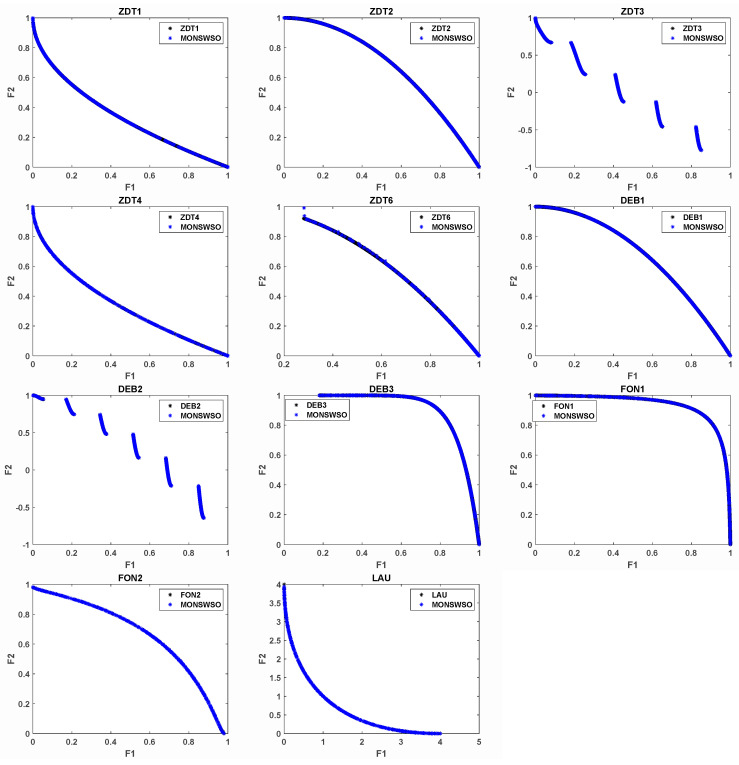
MONSWSO-generated PF and real PF.

Figure 6 presents a comparative illustration of the actual PF achieved by MONSWSO on the two-target test function, juxtaposed against the resultant PF. Figure 6 demonstrates that MONSWSO can swiftly and efficiently approximate the genuine PF of the problem.

#### 4.2.2. Three-Objective Test Problems

Table 4 presents the M and Sd of the four metrics, along with the test results obtained on the three-objective problems. The bold is to emphasize the best value.

In terms of convergence accuracy, MONSWSO performs less effectively than NSGAII and PESAII on WFG4, WFG5, WFG7, and WFG8. However, MONSWSO’s results surpass the comparative algorithms on the other test functions, indicating that MONSWSO possesses good convergence properties.

Regarding the Spacing metric, DTLZ4 falls behind MOPSO, while DTLZ7 lags behind both MOPSO and MOALO. Additionally, WFG2 is inferior to NSGAII, PESAII, MOPSO, MOALO, and MOGWO. WFG4 is also outperformed by NSGAII, PESAII, and MOGWO. Meanwhile, WFG7 is only worse than MOGWO. Notably, MONSWSO outperforms the other algorithms on the remaining functions, demonstrating the algorithm’s good uniformity. Furthermore, according to the Spread metric, MONSWSO achieves the best results across all functions, highlighting its breadth.

From the HV metrics, with the exception of DTLZ6 and WFG6, the metric values for all other test functions fall below those of NSGAII. Furthermore, more than half of these metric values are inferior to those of PESAII. Nevertheless, they generally outperform the remaining comparison algorithms, indicating the potential for enhancing the overall capability of MONSWSO.

Regarding the IGD metrics, MONSWSO exhibited significant differences compared to NSGAII, PESAII, and MOGWO on 4, 6, and 11 out of 12 problems, respectively. Similarly, it demonstrated significant divergence from MOPSO and MOALO on 12 test functions, respectively. In terms of the Spacing metric, MONSWSO showed significant distinctions from NSGAII, PESAII, MOPSO, MOALO, and MOGWO on 7, 8, 5, 10, and 7 test functions, respectively.

When analyzing Spread metrics, NSGAII and PESAII were not notably different from MONSWSO on the DTLZ2 problem, and both were indistinguishable from MOPSO on 5 test functions. As for HV metrics, MONSWSO demonstrated significant variations from NSGAII, PESAII, and MOPSO on 2, 4, and 9 test functions, respectively. Furthermore, MONSWSO exhibited significant differences from both MOALO and MOGWO across all applicable test functions.

**Figure 7:**
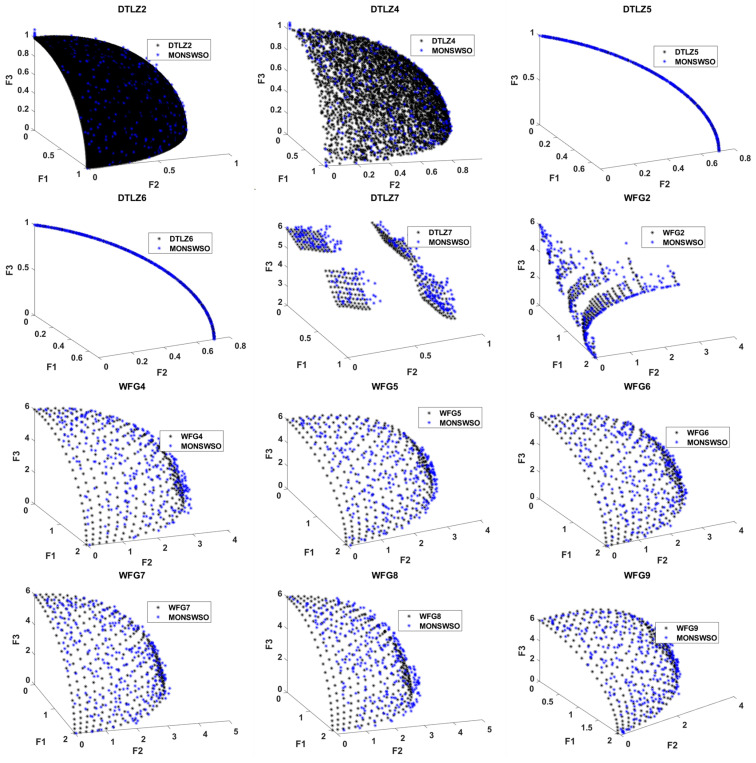
Comparison of MONSWSO-generated Pareto with real Pareto.

Figure 7 plots a comparative representation of the true PF achieved by MONSWSO on the three-objective test function and the obtained PF. Figure 7 illustrates that MONSWSO is equally proficient in efficiently searching for the true PF of the problem, even in the context of a three-objective scenario.

### 4.3. MO Engineering Design Issues

**Figure 8:**
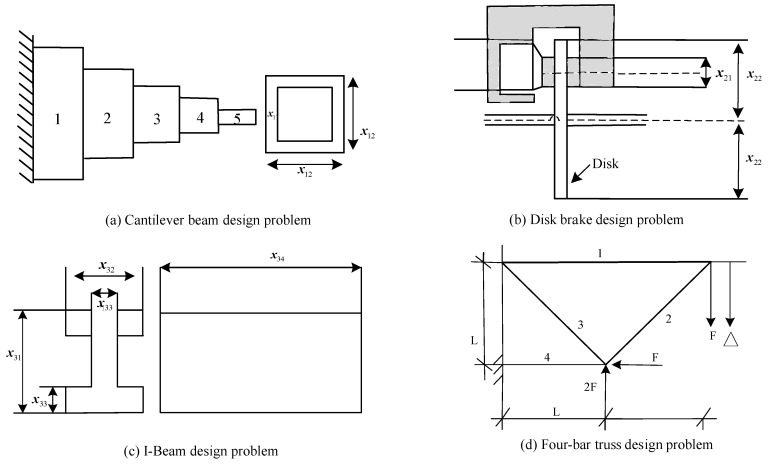
Schematic diagram of the 4 engineering optimization problems.

While the representativeness of MO benchmarking problems poses challenges in validating algorithm performance, the validation of real-world examples holds greater appeal. To assess the utility of MONSWSO, four MO engineering optimization problems were further employed to validate the superior optimization capabilities of MONSWSO. Consistent with the previous section, MONSWSO was independently executed ten times for each instance, and its optimization results were compared with those of NSGAII, MOPSO, PESAII, SMPSO [45], and IBEA [46] using spacing metrics. In this paper, constraints were handled using the penalty function approach. Figure 8a–d present the schematic diagrams of these four engineering optimization problems.

The cantilever beam design [47] problem represented by Figure 8a is an important MO-constrained problem, where the length $x_{11}$ and width $x_{12}$ of the beam are designed to minimize the weight and deflection of the beam under certain constraints.

The disc brake problem [48] represented by Figure 8b is composed of four variables, the meaning of $x_{21},x_{22}$, $x_{23}$, $x_{24}$ are the same as in the literature.

The objective of the I-beam design [49] represented by Figure 8c is to minimize the cross-sectional area and the beam deflection, respectively.

The four-bar truss problem [50,51] represented by Figure 8d aims at optimizing the cross-sectional areas $x_{41}$, $x_{42}$, $x_{43}$, $x_{44}$ of the structural members 1,2,3,4 thereby minimizing the structural weight and nodal displacements.

**Figure 9:**
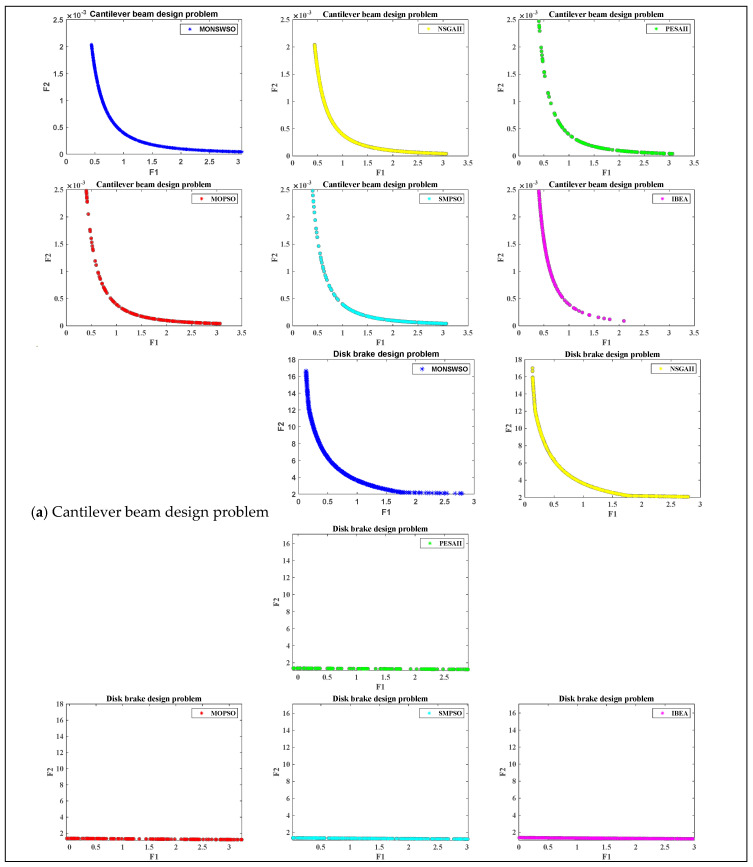

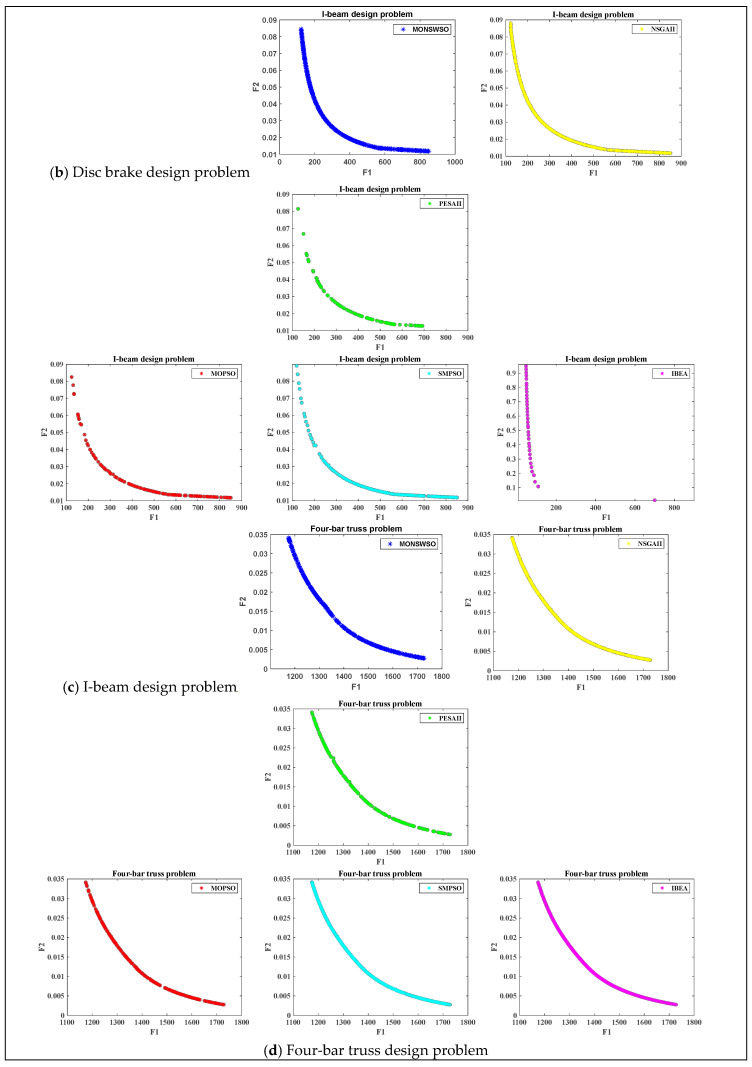
Best Pareto front for each algorithm on four engineering optimization problems.

Table 5 gives the M and Sd of the Spacing metrics for six algorithms for the four engineering instances. The bold is to emphasize the best value. Figure 9a–d show the optimal PF obtained by each algorithm for the four engineering instances.

Table 5 and Figure 9 indicate that the MONSWSO algorithm outperforms the compared methods in terms of solution homogeneity for the real-world instance, achieving continuous Pareto frontiers with excellent solution coverage.

### 4.4. Optimization Design for Foundation Pit Above Metro Tunnel

Subway tunnels situated above large pit excavations will alter the stress field surrounding the tunnels, leading to upward displacement. Significant vertical displacement can have a profound impact on the durability of subway tunnels, not only increasing operational costs but also compromising subway safety. The focal point of subway tunnel pit optimization design [52] lies in devising solutions for the MO optimization problem, which aims to minimize vertical displacement and project costs. This is achieved by considering the number of longitudinal excavation blocks $x_{1}$, the thickness of the anti-floating plate $x_{2}$, the length of anti-piling $x_{3}$, the diameter of anti-piling $x_{4}$,and the number of unilateral anti-piling $x_{5}$ as decision-making variables. These variables collectively form the basis for the optimization design model of the pit.

(21) $$\begin{array}{ll} \min\;f_{1}\left(x\right)= & 17.01\cdot (-0.0000131849x_{1}^{5}+0.000469463x_{1}^{4}-0.00604901x_{1}^{3}+\\ & 0.0343602x_{1}^{2}-0.00992232x_{1}+1.26013)\cdot (-00.01459x_{2}^{5}+\\ & 0.0959586x_{2}^{4}-0.212419x_{2}^{3}+0.199501x_{2}^{2}-0.231241x_{2}+\\ & 1.1189)\times (0.00000216142x_{3}^{5}-0.000113656x_{3}^{4}+\\ & 0.00217459x_{3}^{3}-0.018897x_{3}^{2}+0.0466485x_{3}+\\ & 1.32445)\cdot (-0.033511x_{4}+1.03334)\times \\ & \left(-0.0141426x_{5}+1.39165\right) \end{array}$$

(22) $$\begin{array}{l} \min\;f_{2}\left(x\right)=(119850+4500\cdot \frac{80.5}{x_{1}})\cdot x_{1}+1638175x_{2}+450\pi x_{4}^{2}x_{3}x_{5}+500\pi x_{4}^{2}x_{5} \end{array}$$

where $f_{1}\leq 20mm$, $1\leq x_{1}\leq 13$, $x_{1}\in N$, $0\leq x_{2}\leq 2.8$, $x_{2}\in 0.1\times N$, $3\leq x_{3}\leq 21$, $x_{3}\in 0.5\times N$, $0.6\leq x_{4}\leq 1.6$, $x_{4}\in 0.05\times N$, $6\leq x_{4}\leq 11$, $x_{5}\in N$.

The results obtained by MONSWSO, NSGAII, PESAII, MOPSO, SMPSO, IBEA, MOALO, and MOGWO are rigorously evaluated using the Spacing and HV indicators. The first two solutions, characterized by the optimal objective value and decision variable value, with the greatest distance from the congestion degree, are listed in Table 6. Table 7 presents the M, Sd, optimal value (Best), and median value (Mid) for each algorithm, in relation to both the Spacing and HV indicators.

**Figure 10:**
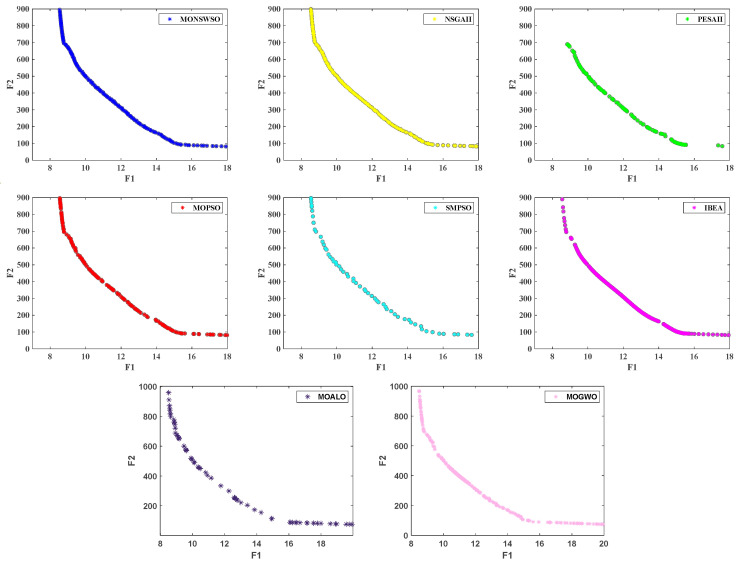
PF obtained by each algorithm.

**Figure 11:**
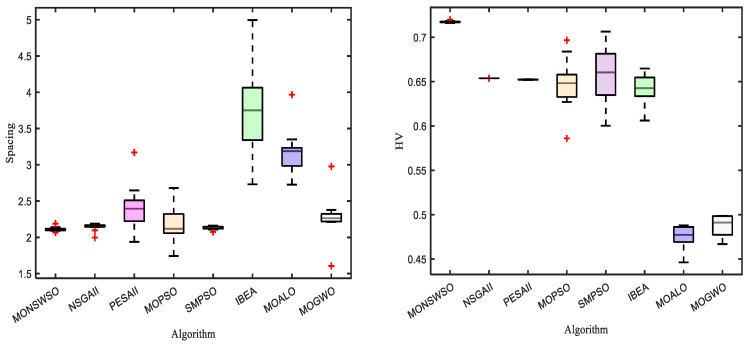
Box plots of Spacing and HV metrics for each algorithm.

The PF and the box-and-line diagram are depicted in Figure 10 and Figure 11, respectively. Notably, Table 7 demonstrates that MONSWSO achieves optimal performance in terms of both Spacing and HV metrics. The bold is to emphasize the best value. Furthermore, Figure 10 and Figure 11 vividly illustrate that MONSWSO provides a satisfactory solution with exceptional uniformity and stability.

## 5. Conclusions

The MONSWSO algorithm seamlessly integrates the WSO with NDS and CD to enhance its performance significantly. It employs a chaotic inverse initialization strategy to generate a high-quality initial population, thereby improving both the uniformity of population distribution and the adaptability of solutions. A variable evolution scheme is utilized to bolster the algorithm’s local exploitation capabilities, while stochastic wandering and vortex effects further enhance the exploration of potential regions, enabling a rapid convergence to the problem’s PF. As a novel MO optimization method, MONSWSO demonstrates notable advantages.

MONSWSO achieves superior uniformity, extensiveness, and comprehensiveness in generating Pareto solutions. The enhancements in uniformity and extensiveness are attributed to the hybrid initialization strategy, energy switching strategy, and adaptive evolutionary approach. The comprehensive performance and solution coverage are further bolstered by non-dominated ordering, crowding distance, and elite selection mechanisms.

When applied to optimize instances, MONSWSO demonstrates a broader range of informative solutions. However, it also has certain limitations; specifically, its performance on two-objective problems outshines that on three-objective problems. Therefore, further refinement of the algorithm is required to address more complex three-objective challenges. The nature of NDS can sometimes lead to a large number of non-dominated solutions generated during iterations, negatively impacting convergence performance. Future research should focus on designing WSO-based MO algorithms that can effectively solve problems with three or more objectives, as well as their application to high-dimensional network optimization problems. Additionally, integrating machine learning techniques to predict and guide the search process has the potential to significantly boost efficiency. Scalability is another critical aspect, as real-world problems often involve a large number of objectives and variables, necessitating robust and scalable algorithms.

## Figures and Tables

**Table 1:** Validation of 23 MO problems.

Function	ZDT1-ZDT4	ZDT6	DEB1-DEB3	FON1-FON2	LAU	DTLZ2, DTLZ4-7	WFG2, WFG4-9
Targets	2	−2	2	2	2	3	3
Dimensions	30	10	2	2	2	12	12
Variable range	[0,1]	[0,1]	[0,1]	[−4,4]	[−50,50]	[0,1]	[0,2:2:24]

**Table 2:** Description of 4 engineering problems.

Function	Four-Bar Truss	Cantilever Beam	Disk Brake	I Beam
Targets	2	−2	2	2
Dimensions (constrain)	4 (0)	2 (2)	4 (5)	4 (1)

**Table 3:** Comparison of MONSWSO indicators on two objective test functions.

	Algorithms	MONSWSO	NSGAII	PESAII	MOPSO	MOALO	MOGWO
Indicators		M (Sd)	M (Sd)	M (Sd)	M (Sd)	M (Sd)	M (Sd)
ZDT1	IGD	**0.00132 (0.00006)**	0.00223 (0.00024) −	0.0049 (0.00064) −	0.30310 (0.0049) −	0.23260 (0.03573) −	0.00595 (0.00542) −
	Spacing	**0.00224 (0.00010)**	0.00321 (0.01786) −	0.00439 (0.00074) −	0.00582 (0.00062) −	0.00232 (0.00278) −	0.00517 (0.00438) −
	Spread	**0.35420 (0.01856)**	0.47234 (0.04477) −	0.98300 (0.04530) −	0.85800 (0.04300) −	1.09400 (0.03887) −	1.26600 (0.12940) −
	HV	0.7229 (0.00004)	**0.7229 (0.00002) +**	0.36316 (0.17000) =	0.71922 (0.00122) −	0.51140 (0.02140) −	0.70600 (0.00651) −
ZDT2	IGD	**0.00201 (0.00052)**	0.01990 (0.09580) −	0.00592 (0.00099) −	0.47000 (0.50800) −	0.58610 (0.01956) −	0.00616 (0.00496) −
	Spacing	**0.00233 (0.00034)**	0.00582 (0.01260) −	0.00445 (0.00096) −	0.00582 (0.00367) −	0.00319 (0.00017) −	0.00618 (0.00456) −
	Spread	**0.35250 (0.02730)**	0.42000 (0.11200) −	0.92000 (0.05020) −	0.86500 (0.11000) −	1.00200 (0.00225) −	1.16200 (0.17660) −
	HV	**0.44750** (**0.00001**)	0.44721 (0.00003) −	0.43879 (0.00239) −	0.01394 (0.03050) −	0.09167 (0.00129) −	0.41970 (0.00781) −
ZDT3	IGD	**0.00164 (0.00005)**	0.00217 (0.00007) −	0.00390 (0.00119) −	0.32400 (0.11100) −	0.05629 (0.03323) −	0.00436 (0.00480) −
	Spacing	**0.00225** (**0.00014**)	0.00327 (0.00026) −	0.00457 (0.00090) −	0.00680 (0.00165) −	0.00749 (0.00806) −	0.00759 (0.0032) −
	Spread	**0.35960 (0.02214)**	0.40700 (0.02940) −	0.92700 (0.04870) −	0.85000 (0.02960) −	1.31400 (0.16610) −	1.11600 (0.06785) −
	HV	0.60070 (0.00004)	0.59064 (0.00003) −	0.60941 (0.02800) +	0.28254 (0.07930) −	**0.66060 (0.05070) +**	0.60810 (0.03257) +
ZDT4	IGD	**0.00150 (0.00007)**	0.00302 (0.00055) −	0.00361 (0.00205) −	11.50000 (5.44000) −	0.14828 (0.06440) −	0.14489 (0.00465) −
	Spacing	**0.00217** (**0.00012**)	0.00342 (0.00028) −	0.00480 (0.00105) −	0.00801 (0.00858) =	0.00549 (0.00394) −	0.00363 (0.00527) −
	Spread	**0.36740 (0.02234)**	0.38800 (0.04290) =	0.90800 (0.10400) −	0.98100 (0.01690) −	1.08300 (0.05164) −	1.22140 (0.05273) −
	HV	**0.73150** (**0.00006**)	0.71276 (0.00026) −	0.71550 (0.00202) −	NaN (NaN) −	0.55400 (0.04146) −	0.70630 (0.00533) −
ZDT6	IGD	**0.00135 (0.00049)**	0.00193 (0.00008) −	0.00274 (0.00040) −	0.00265 (0.02600) −	0.01759 (0.01080) −	0.00445 (0.04722) −
	Spacing	0.00337 (0.00537)	**0.00283 (0.00019)** +	0.04880 (0.00064) =	0.01890 (0.01720) −	0.00752 (0.01445) −	0.01107 (0.01149) −
	Spread	**0.29960 (0.11480)**	0.38400 (0.02780) =	1.09000 (0.31100) −	1.01000 (0.20600) −	1.58100 (0.11970) −	1.05300 (0.07541) −
	HV	**0.40980 (0.00071)**	0.39070 (0.00004) −	0.38751 (0.00059) −	0.38212 (0.01423) −	0.34680 (0.01225) −	0.36670 (0.00838) −
DEB1	IGD	0.00186 (0.00054)	**0.00162 (0.00005)** −	0.00277 (0.00022) −	0.00477 (0.00030) −	0.02599 (0.00581) −	0.00584 (0.00307) −
	Spacing	**0.00227 (0.00028)**	0.00251 (0.00009) −	0.00373 (0.00026) −	0.00601 (0.00062) −	0.00509 (0.00386) −	0.00622 (0.00433) −
	Spread	**0.34620 (0.03558)**	0.42300 (0.03140) −	0.82300 (0.04650) −	0.91800 (0.04620) −	1.22200 (0.10150) −	1.16000 (0.06317) −
	HV	**0.44750 (0.00012)**	0.44236 (0.00005) −	0.44350 (0.00110) −	0.44608 (0.00025) −	0.39830 (0.00383) −	0.42210 (0.00494) −
DEB2	IGD	**0.00164 (0.00011)**	0.13600 (0.00002) −	0.15600 (0.00003) −	0.15600 (0.00044) −	0.02847 (0.01877) −	0.00542 (0.00452) −
	Spacing	**0.00211 (0.00005)**	0.00269 (0.00014) −	0.00412 (0.00031) −	0.00447 (0.00030) −	0.00454 (0.01530) −	0.00766 (0.00989) −
	Spread	**0.37490 (0.01518)**	0.60800 (0.03400) −	0.90200 (0.02830) −	1.15000 (0.03610) −	1.33100 (0.29300) −	1.16700 (0.12040) −
	HV	**0.47640 (0.00002)**	0.45039 (0.00001) −	0.44994 (0.00007) −	0.45020 (0.00048) −	0.44570 (0.01793) −	0.45660 (0.00541) −
DEB3	IGD	**0.00159 (0.00016)**	0.00524 (0.00094) −	0.00763 (0.00092) −	0.00928 (0.00653) −	0.03210 (0.02386) −	0.00710 (0.02386) −
	Spacing	**0.00197 (0.00013)**	0.00664 (0.00054) −	0.00867 (0.00071) −	0.00848 (0.00073) −	0.00446 (0.00506) −	0.00616 (0.00506) −
	Spread	**0.34710 (0.02220)**	0.42400 (0.05370) −	0.83400 (0.07320) −	0.74900 (0.05960) −	1.35800 (0.17400) −	1.01800 (0.17400) −
	HV	**0.24310 (0.00010)**	0.23158 (0.00011) −	0.22980 (0.00075) −	0.22943 (0.00237) −	0.20430 (0.00631) −	0.21700 (0.00626) −
FON1	IGD	**0.00203 (0.00019)**	0.00284 (0.00006) −	0.00335 (0.00024) −	0.00293 (0.00018) **−**	0.05434 (0.01743) **−**	0.07805 (0.00944) **−**
	Spacing	0.00242 (0.00038)	0.00284 (0.00010) −	0.00375 (0.00024) −	0.00328 (0.00018) −	**0.00145 (0.00454) +**	0.01701 (0.00235) −
	Spread	**0.36340 (0.02442)**	0.41600 (0.02910) −	0.89600 (0.03140) −	0.75500 (0.02730) −	1.07200 (0.12220) +	1.04600 (0.12670) −
	HV	**0.22590 (0.00001)**	0.22585 (0.00003) −	0.22409 (0.00056) −	0.22544 (0.00019) −	0.18950 (0.00964) −	0.21190 (0.00371) −
FON2	IGD	0.00216 (0.00023)	**0.00201 (0.00015) +**	0.00482 (0.00115) **−**	0.00356 (0.00057) **−**	0.04959 (0.01091) **−**	0.01508 (0.00407) **−**
	Spacing	**0.00232 (0.00003)**	0.00239 (0.00008) −	0.00407 (0.00038) −	0.00354 (0.00035) −	0.00294 (0.00379) −	0.00412 (0.00159) −
	Spread	**0.35780 (0.02195)**	0.41000 (0.02840) −	0.92500 (0.02970) −	0.81000 (0.03920) −	1.03500 (0.11720) −	0.86640 (0.01767) −
	HV	**0.43130 (0.00006)**	0.42085 (0.00007) −	0.42570 (0.00163) −	0.42965 (0.00062) −	0.38510 (0.00695) −	0.41080 (0.00252) −
LAU	IGD	0.00688 (0.00065)	**0.00674 (0.00076) +**	0.01560 (0.00161) −	0.01190 (0.00163) −	0.14070 (0.03893) −	0.02493 (0.02529) −
	Spacing	**0.00832 (0.00040)**	0.01030 (0.00048) −	0.01690 (0.00258) −	0.01470 (0.00065) −	0.04747 (0.01946) −	0.03520 (0.01049) −
	Spread	**0.34570 (0.02212)**	0.48300 (0.03310) −	1.01000 (0.05430) −	0.82600 (0.02760) −	1.37100 (0.10770) −	1.15600 (0.13540) −
	HV	**0.88100 (0.00002)**	0.76107 (0.00001) −	0.85937 (0.00037) −	0.81007 (0.00013) −	0.84080 (0.00355) −	0.85330 (0.00262) −
+/−/=	IGD		2/9/0	0/11/0	0/11/0	1/10/0	0/11/0
	Spacing		1/10/0	0/10/0	0/10/0	1/10/0	0/11/0
	Spread		0/9/2	0/11/0	0/11/0	0/11/0	0/11/0
	HV		1/10/0	1/9/1	0/11/0	1/10/0	1/10/0

**Table 4:** Comparison of MONSWSO indicators on the three objective test functions.

	Algorithms	MONSWSO	NSGAII	PESAII	MOPSO	MOALO	MOGWO
Indicators		M (Sd)	M (Sd)	M (Sd)	M (Sd)	M (Sd)	M (Sd)
DTLZ2	IGD	**0.03697 (0.00042)**	0.03910 (0.00094) =	0.03930 (0.00062) =	0.04840 (0.00346) −	0.11950 (0.01543) −	0.09500 (0.02922) −
	Spacing	**0.03002 (0.00228)**	0.03210 (0.00108) −	0.03290 (0.00112) −	0.03130 (0.00175) −	0.06412 (0.00824) −	0.03458 (0.00169) −
	Spread	**0.34650 (0.00312)**	0.50135 (0.01760) =	0.55242 (0.03930) =	0.38481 (0.02241) =	1.36412 (0.00824) −	0.55458 (0.00169) −
	HV	0.54650 (0.00310)	**0.56335 (0.00265) +**	0.56312 (0.00195) +	0.54534 (0.00926) −	0.40042 (0.02112) −	0.43421 (0.03326) −
DTLZ4	IGD	**0.03851 (0.00067)**	0.03920 (0.00069) =	0.03959 (0.00069) −	0.14384 (0.10000) −	0.36850 (0.01417) −	0.09210 (0.02972) −
	Spacing	0.02868 (0.00148)	0.03260 (0.00127) =	0.03332 (0.00085) −	**0.02575 (0.01460) +**	0.03810 (0.01984) −	0.03430 (0.00246) −
	Spread	**0.39160 (0.02081)**	0.51175 (0.02330) −	0.53451 (0.03280) −	0.55518 (0.13900) −	1.47900 (0.08540) −	0.81820 (0.02073) −
	HV	0.56371 (0.00168)	0.56511 (0.00151) +	**0.56752 (0.00192) +**	0.48967 (0.04455) =	0.29450 (0.06226) −	0.17904 (0.01743) −
DTLZ5	IGD	**0.00181 (0.00011)**	0.00189 (0.00007) =	0.00412 (0.00042) −	0.00411 (0.00040) −	0.03650 (0.02382) −	0.01494 (0.01102) −
	Spacing	**0.00277** (**0.00008**)	0.00302 (0.00022) =	0.00568 (0.00081) −	0.00543 (0.00054) −	0.02064 (0.01341) −	0.00711 (0.00188) −
	Spread	**0.38140 (0.01083)**	0.43658 (0.04740) −	0.92704 (0.04120) −	0.95067 (0.06150) −	1.38600 (0.12390) −	1.17800 (0.08354) −
	HV	0.20142 (0.00003)	**0.20157 (0.000001) +**	0.19886 (0.00135) =	0.19771 (0.00165) =	0.13895 (0.01752) −	0.1669 (0.02245) −
DTLZ6	IGD	**0.00165 (0.00014)**	0.00188 (0.00005) −	0.00462 (0.00027) −	1.77190 (0.87200) −	0.05422 (0.04962) −	0.00373 (0.00136) −
	Spacing	**0.00283** (**0.00011**)	0.00357 (0.00016) −	0.00531 (0.00052) −	0.06821 (0.02460) −	0.08127 (0.05243) −	0.00391 (0.00148) −
	Spread	**0.40600 (0.02047)**	0.61763 (0.03520) −	1.16700 (0.04690) −	0.59714 (0.07260) −	1.46300 (0.20750) −	0.86790 (0.07747) −
	HV	**0.20173 (0.00006)**	0.20162 (0.00003) −	0.19909 (0.00074) −	NAN (NAN) −	0.16622 (0.01276) −	0.1921 (0.00822) −
DTLZ7	IGD	**0.03978 (0.00311)**	0.04108 (0.00230) =	0.04209 (0.00223) =	0.72841 (0.39800) −	0.57880 (0.02365) −	0.04730 (0.06067) =
	Spacing	0.03029 (0.00217)	0.03808 (0.00345) −	0.03496 (0.00205) −	0.01827 (0.01090) **+**	**0.01196 (0.00506) +**	0.03877 (0.00791) −
	Spread	**0.44560 (0.01032)**	0.48693 (0.02530) −	0.58002 (0.04870) −	0.57881 (0.16200) −	1.09600 (0.04316) −	0.70110 (0.06978) −
	HV	0.21452 (0.00195)	**0.28210 (0.00058) +**	0.28087 (0.00129) +	0.12391 (0.07210) −	0.16390 (0.02612) −	0.10900 (0.03344) −
WFG2	IGD	**0.11830 (0.00380)**	0.12433 (0.00621) −	0.12430 (0.00710) −	0.17405 (0.01980) −	0.28800 (0.02969) −	0.15840 (0.01953) −
	Spacing	0.14530 (0.01960)	0.12909 (0.04000) +	0.10783 (0.00670) +	**0.09866 (0.04640) +**	0.14490 (0.01973) +	0.11460 (0.02953) **+**
	Spread	**0.36950 (0.01541)**	0.47087 (0.02160) −	0.52292 (0.04230) −	0.44214 (0.02980) −	1.08400 (0.09980) −	0.47540 (0.03116) −
	HV	0.93256 (0.00191)	**0.93443 (0.00081) +**	0.93132 (0.00182) −	0.86914 (0.01842) −	0.82560 (0.02620) −	0.8645 (0.00572) −
WFG4	IGD	0.21010 (0.00484)	**0.16118 (0.00246) +**	0.16457 (0.00337) **+**	0.22022 (0.00712) −	0.61810 (0.10020) −	0.38840 (0.23370) −
	Spacing	0.12400 (0.00762)	0.12022 (0.00626) +	**0.11795 (0.00625) +**	0.12415 (0.00837) =	0.17380 (0.02253) −	0.13520 (0.03277) **+**
	Spread	**0.39918 (0.00729)**	0.42288 (0.02560) −	0.42300 (0.02340) −	0.40542 (0.03290) −	1.49400 (0.03258) −	0.52890 (0.03700) −
	HV	0.5175 (0.00176)	**0.55013 (0.00212) −**	0.54642 (0.00310) −	0.49404 (0.00439) −	0.37492 (0.02349) −	0.3317 (0.02252) −
WFG5	IGD	0.19690 (0.00326)	**0.18070 (0.00334) +**	0.18195 (0.00323) **+**	0.20280 (0.01350) −	0.39650 (0.05902) −	0.49797 (0.15670) −
	Spacing	**0.10710** (**0.00505**)	0.11930 (0.00541) −	0.12474 (0.00949) −	0.11514 (0.00817) −	0.19550 (0.01606) −	0.12720 (0.02631) −
	Spread	**0.36950 (0.01541)**	0.47087 (0.02160) −	0.52292 (0.04230) −	0.44214 (0.02980) −	1.08400 (0.09980) −	0.47540 (0.03116) −
	HV	0.50263 (0.00253)	**0.51788 (0.00317) +**	0.49825 (0.00531) −	0.46605 (0.00881) −	0.41842 (0.02600) −	0.27884 (0.01552) −
WFG6	IGD	**0.16722 (0.00433)**	0.20847 (0.00874) −	0.19431 (0.01260) −	0.23406 (0.02620) −	0.60000 (0.07342) −	0.47700 (0.07941) −
	Spacing	**0.11364** (**0.00617**)	0.12953 (0.00768) −	0.12812 (0.00712) =	0.17190 (0.00677) −	0.20220 (0.02319) −	0.15830 (0.02722) −
	Spread	**0.38990 (0.01927)**	0.49648 (0.02690) −	0.52410 (0.01850) −	0.40585 (0.02080) =	1.58300 (0.03202) −	0.62880 (0.04042) −
	HV	**0.54564 (0.00222)**	0.49859 (0.00952) −	0.50025 (0.01250) −	0.46985 (0.00964) −	0.33832 (0.0263) −	0.26622 (0.01669) −
WFG7	IGD	0.16956 (0.00359)	**0.16431 (0.00348) +**	0.16579 (0.00223) **+**	0.24305 (0.01340) −	0.57120 (0.05648) −	0.61830 (0.08866) −
	Spacing	0.11299 (0.00376)	0.12813 (0.00938) =	0.12793 (0.00662) =	0.11785 (0.00805) =	0.19230 (0.01449) −	**0.10970 (0.03292) +**
	Spread	**0.39097 (0.01326)**	0.53448 (0.02520) −	0.54623 (0.03090) −	0.41414 (0.02160) =	1.46500 (0.02506) −	0.61280 (0.04507) −
	HV	0.54378 (0.00242)	**0.56474 (0.00128) +**	0.54612 (0.00403) +	0.47741 (0.00639) −	0.35372 (0.00930) −	0.26422 (0.01316) −
WFG8	IGD	0.27467 (0.00301)	0.27150 (0.00373) **+**	**0.25909 (0.00557) +**	0.39959 (0.01510) −	0.75280 (0.07509) −	0.66374 (0.15700) −
	Spacing	**0.11605** (**0.00352**)	0.13303 (0.00665) −	0.13346 (0.00661) −	0.12294 (0.00562) =	0.17890 (0.02275) −	0.14190 (0.02361) =
	Spread	**0.40327 (0.01505)**	0.54412 (0.02860) −	0.55864 (0.03350) −	0.40969 (0.02190) =	1.55200 (0.03676) −	0.58990 (0.03998) −
	HV	0.53927 (0.00220)	**0.56474 (0.00128) +**	0.54612 (0.00403) **+**	0.47741 (0.00639) −	0.30262 (0.00840) −	0.19697 (0.02371) −
WFG9	IGD	**0.15622 (0.00445)**	0.16649 (0.00399) −	0.16057 (0.00172) −	0.18701 (0.01120) −	0.46700 (0.06412) −	0.54952 (0.25570) −
	Spacing	**0.10778** (**0.00371**)	0.11692 (0.00481) −	0.11654 (0.00549) −	0.11812 (0.00448) −	0.18730 (0.01528) −	0.12890 (0.02646) −
	Spread	**0.38916 (0.01534)**	0.46482 (0.02810) −	0.44877 (0.03030) −	0.40412 (0.02940) =	1.37900 (0.27080) −	0.59610 (0.03859) −
	HV	0.46776 (0.00393)	**0.53804 (0.00174) +**	0.52620 (0.00255) **+**	0.50723 (0.00739) **+**	0.42399 (0.03350) −	0.26681 (0.02237) −
+/−/=	IGD		4/4/4	4/6/2	0/12/0	0/12/0	0/11/1
	Spacing		2/7/3	2/8/2	3/5/4	2/10/0	3/7/2
	Spread		0/11/1	0/11/1	0/7/5	0/12/0	0/12/0
	HV		10/2/0	7/4/1	1/9/2	0/12/0	0/12/0

**Table 5:** Spacing metric results for four engineering optimization problems.

Algorithm	Spacing							
	Problem a		Problem b		Problem c		Problem d	
	M	Sd	M	Sd	M	Sd	M	Sd
MONSWSO	**0.00427**	0.00032	**0.01752**	**0.00051**	**1.11920**	**0.01200**	0.87604	0.04870
NSGAII	0.00560	**0.00012**	0.02985	0.01010	4.26730	0.40300	0.88125	0.05470
PESAII	0.01014	0.00096	NaN	NaN	5.42750	0.86800	1.19350	0.09310
MOPSO	0.00926	0.00082	NaN	NaN	5.89620	1.81000	1.16510	0.09820
SMPSO	0.00669	0.00065	NaN	NaN	6.00830	0.73800	**0.86004**	0.04320
IBEA	0.04012	0.01533	NaN	NaN	NaN	NaN	1.07990	**0.03230**

**Table 6:** Optimized design results of the pit above the subway tunnel.

Algorithm	Problem Design Objectives		Problem Design Parameters				
	$f_{1}$	$f_{2}$	$x_{1}$	$x_{2}$	$x_{3}$	$x_{4}$	$x_{5}$
MONSWSO	12.31259	282.03516	10	0.5	20	0.6	41
	13.27690	237.44859	5	0.6	19.5	0.6	41
NSGAII	12.95089	251.52024	6	0.6	20.5	0.6	41
	12.45820	275.49024	8	0.6	20.5	0.6	41
PESAII	11.93554	315.12135	10	0.7	20	0.6	41
	11.95881	313.31412	10	0.7	20.5	0.6	41
MOPSO	11.89600	320.23870	9	0.8	20.5	0.6	41
	11.40560	363.94390	10	1	20	0.6	41
SMPSO	11.52850	351.95890	9	1	20	0.6	41
	12.96330	276.69480	7	0.7	20	0.6	39
IBEA	12.62490	253.66840	9	0.4	20	0.6	41
	12.56850	284.28370	6	0.8	20.5	0.6	41
MOALO	11.80487	332.80187	8	0.9	20	0.6	41
	12.54890	267.25680	8	0.5	20	0.6	41
MOGWO	12.55031	260.80876	9	0.4	21	0.6	41
	12.45241	294.43582	6	0.8	20.5	0.6	41

**Table 7:** Spacing and HV results.

Algorithm	Spacing				HV			
	M	Sd	Best	Mid	M	Sd	Best	Mid
MONSWSO	**2.11610**	**0.03834**	**2.06270**	**2.10952**	**0.71729**	0.00112	**0.71993**	**0.71040**
NSGAII	2.14021	0.10221	2.08900	2.13762	0.65374	**0.00001**	0.65411	0.65374
PESAII	2.31313	0.38521	2.11154	2.30851	0.65224	0.00029	0.65350	0.65219
MOPSO	2.16012	0.36815	2.07235	2.17433	0.65263	0.05120	0.69272	0.65277
SMPSO	2.11922	0.04162	2.07525	2.12742	0.65383	0.04160	0.70644	0.65364
IBEA	3.79824	0.55214	3.24743	3.55814	0.64373	0.02110	0.65385	0.64373
MOALO	3.18780	0.50137	2.73218	3.32476	0.47226	0.00601	0.48693	0.47108
MOGWO	2.26113	0.41588	2.19739	2.23096	0.48691	0.00082	0.48868	0.48729

## Data Availability

Data will be made available on request.

## Appendix A

**Table A1:** The meaning of abbreviations.

Abbreviation	Meaning
MO	multi-objective
WSO	White Shark Optimization algorithm
NDS	non-dominated sorting
CD	crowding distance
IGD	inverse generation distance
Spacing	spatial homogeneity
Spread	spatial distribution
HV	hypervolume
PF	pareto front
MONSWSO	multi-objective White Shark Optimization algorithm
WCs	weight vectors
POS	Pareto optimal solutions
